# Supplementary material for: Carotid body dysregulation contributes to Long COVID symptoms
Source: Commun Med (Lond). 2024 Feb 19;4:20. doi: 10.1038/s43856-024-00447-5 (PMC10876702; doi:10.1038/s43856-024-00447-5)
Supplement: Supplementary file 5 — Description of Additional Supplementary Files [file 43856_2024_447_MOESM5_ESM.docx]

**Description of Additional Supplementary Files**

**File Name:** Supplementary Data 1

**Description:** Raw data used to make Figure 1

**File Name:** supplementary Data 2

**Description:** Data underlying Figure 2
