# Supplementary material for: Carotid body dysregulation contributes to Long COVID symptoms
Source: Commun Med (Lond). 2024 Feb 19;4:20. doi: 10.1038/s43856-024-00447-5 (PMC10876702; doi:10.1038/s43856-024-00447-5)
Supplement: Supplementary file 6 — Reporting Summary [file 43856_2024_447_MOESM6_ESM.pdf]

nature portfolio

Corresponding author(s): Emma C Hart

Last updated by author(s): Oct 11, 2023

Reporting Summary

Nature Portfolio wishes to improve the reproducibility of the work that we publish. This form provides structure for consistency and transparency in reporting. For further information on Nature Portfolio policies, see our Editorial Policies and the Editorial Policy Checklist.

Statistics

For all statistical analyses, confirm that the following items are present in the figure legend, table legend, main text, or Methods section.

n/a	Confirmed	
☐	☒	The exact sample size (*n*) for each experimental group/condition, given as a discrete number and unit of measurement
☐	☒	A statement on whether measurements were taken from distinct samples or whether the same sample was measured repeatedly
☐	☒	The statistical test(s) used AND whether they are one- or two-sided<br>*Only common tests should be described solely by name; describe more complex techniques in the Methods section.*
☐	☒	A description of all covariates tested
☐	☒	A description of any assumptions or corrections, such as tests of normality and adjustment for multiple comparisons
☐	☒	A full description of the statistical parameters including central tendency (e.g. means) or other basic estimates (e.g. regression coefficient) AND variation (e.g. standard deviation) or associated estimates of uncertainty (e.g. confidence intervals)
☐	☒	For null hypothesis testing, the test statistic (e.g. *F*, *t*, *r*) with confidence intervals, effect sizes, degrees of freedom and *P* value noted<br>*Give P values as exact values whenever suitable.*
☒	☐	For Bayesian analysis, information on the choice of priors and Markov chain Monte Carlo settings
☒	☐	For hierarchical and complex designs, identification of the appropriate level for tests and full reporting of outcomes
☒	☐	Estimates of effect sizes (e.g. Cohen's *d*, Pearson's *r*), indicating how they were calculated

Our web collection on statistics for biologists contains articles on many of the points above.

Software and code

Policy information about availability of computer code

Data collection	N/A
Data analysis	N/A

For manuscripts utilizing custom algorithms or software that are central to the research but not yet described in published literature, software must be made available to editors and reviewers. We strongly encourage code deposition in a community repository (e.g. GitHub). See the Nature Portfolio guidelines for submitting code & software for further information.

Data

Policy information about availability of data

All manuscripts must include a data availability statement. This statement should provide the following information, where applicable:

- Accession codes, unique identifiers, or web links for publicly available datasets
- A description of any restrictions on data availability
- For clinical datasets or third party data, please ensure that the statement adheres to our policy

Source data underlying Figure 1 and 2 are provided as Supplementary Data 1 and Supplementary Data 2 excel files. It is the authors' intention to share their underpinning research data to maximise reuse and evidence their findings. At the time these data were generated, participants were not asked for their permission to share data beyond the immediate project team. The data will be deposited at the University of Bristol Research Data Repository (data.bris.ac.uk/data) where once published, they will be assigned the following doi: DOI 10.5523/bris.2w4fl5nhvpl782hhnybwsv3zw. A metadata record will be published openly by the

repository and this record will clearly state how data can be accessed by bona fide researchers. Requests for access will be directed to the Research Data team at Bristol, who will assess the motives of potential data re-users before granting access to the data. No authentic request for access will be refused and re-users will not be charged for any part of this process.

Human research participants

Policy information about studies involving human research participants and Sex and Gender in Research.

Reporting on sex and gender	Sex was determined by self-reporting. All participants self-reported that the they were cis-gender.
Population characteristics	Healthy controls: age=35 ± 13, Long-COVID: age=42 ± 12 years (mean+/-SD).<br>Long-COVID: diagnosed with long-COVID by health care provider, no other known health conditions.
Recruitment	Participants (controls and long-COVID) were recruited by advertisement - email, posters, leaflets. Participants attending long-COVID clinics were told about the study and asked to email/phone the study team if they were interested in participating. Participants meeting the exclusion criteria were excluded.
Ethics oversight	HRA/NHS

Note that full information on the approval of the study protocol must also be provided in the manuscript.

Field-specific reporting

Please select the one below that is the best fit for your research. If you are not sure, read the appropriate sections before making your selection.

☒ Life sciences ☐ Behavioural & social sciences ☐ Ecological, evolutionary & environmental sciences

For a reference copy of the document with all sections, see nature.com/documents/nr-reporting-summary-flat.pdf

Life sciences study design

All studies must disclose on these points even when the disclosure is negative.

Sample size	*Describe how sample size was determined, detailing any statistical methods used to predetermine sample size OR if no sample-size calculation was performed, describe how sample sizes were chosen and provide a rationale for why these sample sizes are sufficient.*
Data exclusions	No data were excluded
Replication	We have not attempted to reproduce the findings presented because this would be an excessive burden to participants (3 more visits and 1 more exercise test which exacerbates long-COVID symptoms for some patients). Also - this would mean we deviate from our ethics protocol.
Randomization	Patients were identified as having long-COVID - they were allocated into the long-COVID. People who had COVID-19 but did not develop ongoing symptoms were allocated to the healthy control group.
Blinding	Investigators were blinded to group allocation during data analysis. It was impossible to blind during data collection due to the patient discussion their symptoms.

Reporting for specific materials, systems and methods

We require information from authors about some types of materials, experimental systems and methods used in many studies. Here, indicate whether each material, system or method listed is relevant to your study. If you are not sure if a list item applies to your research, read the appropriate section before selecting a response.

Materials & experimental systems

n/a	Involved in the study	
☒	☐	Antibodies
☒	☐	Eukaryotic cell lines
☒	☐	Palaeontology and archaeology
☒	☐	Animals and other organisms
☒	☐	Clinical data
☒	☐	Dual use research of concern

Methods

n/a	Involved in the study	
☒	☐	ChIP-seq
☒	☐	Flow cytometry
☒	☐	MRI-based neuroimaging
