# Supplementary material for: Carotid body dysregulation contributes to Long COVID symptoms
Source: Commun Med (Lond). 2024 Feb 19;4:20. doi: 10.1038/s43856-024-00447-5 (PMC10876702; doi:10.1038/s43856-024-00447-5)
Supplement: Supplementary file 4 — Peer Review File [file 43856_2024_447_MOESM4_ESM.pdf]

nature portfolio

Peer Review File

Carotid body dysregulation contributes to Long COVID symptoms

Open Access This file is licensed under a Creative Commons Attribution 4.0 International License, which permits use, sharing, adaptation, distribution and reproduction in any medium or format, as long as you give appropriate credit to the original author(s) and the source, provide a link to the Creative Commons license, and indicate if changes were made. In the cases where the authors are anonymous, such as is the case for the reports of anonymous peer reviewers, author attribution should be to 'Anonymous Referee' followed by a clear attribution to the source work. The images or other third party material in this file are included in the article's Creative Commons license, unless indicated otherwise in a credit line to the material. If material is not included in the article's Creative Commons license and your intended use is not permitted by statutory regulation or exceeds the permitted use, you will need to obtain permission directly from the copyright holder. To view a copy of this license, visit http://creativecommons.org/licenses/by/4.0/.

This manuscript has been previously reviewed at another Nature Portfolio journal. This document only contains reviewer comments and rebuttal letters for versions considered at Communications Medicine.

REVIEWERS' COMMENTS:

Reviewer #2 (Remarks to the Author):

I am satisfied with the authors' responses and the manuscript editions. I have no further concerns.

Reviewer #3 (Remarks to the Author):

Thank you for examining the impact of blood pressure on HVR, and including the VE/VCO2 nadir in your analysis.

The paper is much improved and provides a nice balanced assessment of Long-COVID.

Thank you, and congratulations.
